# Progress in Medicine

**Published:** 1897-07-31

**Authors:** 


### Progress in Medicine.

### DIPHTHERIA.

### Serum Therapy.

**Pathology.**—A very interesting address by W. Whitla[1] on serum-therapy, chiefly in reference to diphtheria, commences with a *résumé* of some facts and theories on the question of immunity. Passing over the subject of natural immunity, Whitla remarks that the discoveries of Metschnikoff at one time seemed to demonstrate that the explanation of acquired immunity lay in the phenomena of phagocytosis, a view that has gradually given way to that first promulgated by Buchner; viz., that acquired immunity or insusceptibility depends upon the presence of certain chemical or unorganised substances or bodies in the blood. He refers to Sir Joseph Lister's statement that Metschnikoff has established the great truth that phagocytosis is the main defensive means possessed by the living body against the invasion of its microscopic foes, and that, especially in the case of natural immunity, phagocytosis seems to be the sole defensive agency. He also believes that in the case of acquired immunity the useful elements of the serum may be in part at least derived from the digestive juices of the phagocytes. Pasteur conceived the idea and carried it into practice of rendering an animal immune by injecting into its tissues gradually increasing doses of attenuated virus. Then followed the demonstration that the animal so treated was proof against enormous doses of the original virus in its most potent form. Whitla, continuing, remarks that Pasteur in this was but working out scientifically the great problem which had been successfully solved by the immortal Jenner many years before. An important advance was made when Salmon demonstrated that an animal could be rendered immune by the injection of gradually increasing doses of the chemical substance manufactured by the bacilli without the injection of any living microbes. Chemical vaccination, in contradistinction to vital or protein vaccination, was then demonstrated to be practical, and gradually it was established that there is no essential difference between the process of immunisation or protection by means of injections of the chemical poison or by small quantities of the living bacilli. Behring found and proved by direct experiment that Pasteurism applied to diphtheria as well as to anthrax and other diseases; but he fortunately went two steps further, and discovered that the blood serum of the protected animal contained chemical substances developed (during the immunisation), which had the power of protecting or immunising another animal if injected into the tissues. This result was found to be true not only in the case of diphtheria, but experiment proved that similar results were obtained with other infective microbes, so that all bacteriologists accept without reservation the law now known as Behring's law, "That if an animal has been artificially protected against a particular infective agent, its blood or serum acquires the power, when injected in sufficient quantity into another animal, of directly transmitting an immunity from that agent" (Kanthack). Behring's final step led to his discovery that the serum or anti-toxin was not only preventive, but curative, and that its administration to an animal or to man already infected with the original microbe possessed distinct antidotal powers.

Whitla then alludes to the nature and origin of the so-called "anti-toxin" in the serum, and remarks that in all probability it will be demonstrated to be a vital product of the living cells of the animal, secreted during the progress of immunisation. Fraser has recently started the theory that, as regards snake-bite, anti-toxin "protection or immunity is chiefly due to the accumulation in the blood of an antidotal substance, and that this substance originates in part, at least, from the venom itself, and is normally one of the constituents of the venom." Buchner had formerly promulgated the same view regarding the anti-toxin of diphtheria. Whitla says that, inasmuch as it must be of the nature of ferment, with the power of reproducing itself (since the serum of a horse prepared for yielding anti-toxin is as strong in anti-toxic power after the second bleeding as after the first), it is highly improbable that Buchner's view is correct; and Calmette denies that Fraser's theory is tenable.

As to how the serum acts, two views hold the field. One is the *chemical* theory, the other theory being that *vital cellular change* rapidly follows upon the injections. The action cannot be explained by the supposition that the serum acts as a poison to the bacilli as corrosive sublimate does, because it has been demonstrated that the bacilli may be cultivated in their respective antitoxic serums. The author recalls the fact that Sydney Martin found two poisons in diphtheria. One he extracted from the diphtheritic membrane as a precipitate; it produces, in one single infinitesimal dose, paralysis and death in the rabbit, preceded by wasting diarrhœa and fatty degeneration; it reproduces the symptoms of pathological changes found in diphtheria after one single minute dose, and is therefore probably a ferment. This poison, which is the chemical representative of the Klebs-Loeffler bacillus, is not found in the blood and tissues of animals or men dying from diphtheria. Then, another poison has been found by Martin, which is an albumose or digested proteid. Injection of this poison produces rapid rise of temperature, and repeated small doses finally cause all the usual characteristics of diphtheria, including the nerve and cardiac degenerations. Martin's view is that the bacilli growing in the membrane produces the toxin or ferment poison, which is absorbed into the blood, where, by acting upon the proteids of the body, it forms the albumose poison, which is thus a product of its digestive action.

Behring's method of determining the anti-toxic value of serums was to mix them in varying quantities with the lethal doses of toxin, and then to administer the mixture to guinea-pigs of 300 grammes weight. When he formed a serum of which 0·1 c.cm. mixed with ten lethal doses could be injected into a guinea-pig without the slightest harm he labelled this serum as possessing one immunising unit. He was able to protect animals to such a degree against diphtheria that one fifteen-thousandth part of 1 c.cm. (= nearly one thousandth part of a drop) of the serum mixed with a fatal dose of diphtheritic toxin, when injected into a guinea-pig, produced no effects whatever. The same dose without the serum always killed the control animal. Further results obtained by Behring bear on the "time" difficulty, which clinical results have so strongly corroborated. It was found that an animal injected with the minimum lethal dose of toxin, and at the same moment with an injection of the serum in some other part of the body, required a dose of serum 100 times more to save it than the quantity necessary when the serum and toxin were previously mixed together before injection. Further, the minimum lethal dose was injected into an animal and thirty minutes allowed to elapse before the serum was administered; the dose of the latter necessary to save the animal was found to be 200 times greater than that used in the test tube experiment. But if double the lethal dose be given to an animal and serum be injected 30 minutes later it does not require such a relatively large dose to save the animal, viz., the amount necessary will fall far short of 400 times the test tube amount, but Professor Fraser insists upon the importance of noting that if the dose of toxin considerably exceeds the minimum lethal dose, possibly for most toxins, when it is as large as three times the minimum lethal, there are probably no existing anti-toxins that are capable of preventing death in any conditions encountered in practice. Whitla says he mentions these latter experiments, because in them the key-note to treatment is to be found, and it is for this reason that we have quoted somewhat fully his very interesting and instructive paper up to this point without going further into the striking clinical results which he analyses and lays before his readers in such a readable and acceptable form.

A striking instance of the influence of school attendance in the spread of diphtheria was mentioned recently by Louis Parkes[2] in a paper read at the Epidemiological Society. It was in June and July, 1896, when of 50 cases of children of school age, 20 were among the girls and infants attending one of the Board schools; the boys, who had a separate playground, with an entrance in another street, remaining exempt.

The influence of fasting and inanition on the action of certain microbial toxins has been investigated by Teissier and Guinard.[3] They found that inanition and fasting result in an increase of the power of resistance to these toxins. A comparative study of well-fed and fasting dogs showed that the effects of pneumo-bacillin and diphtheria toxin are especially influenced by the lack of food. To explain this influence, two theories are forthcoming: Either, in fasting animals in a state of inanition, the toxins find themselves confronted by starved cells, ready to elaborate and assimilate anything that presents itself, and are therefore destroyed before they are able to do all the harm of which they are capable; or the toxins act more slowly, or not at all, because the impoverished organism lacks elements on which their fermentative activity is exerted for the production of rapidly-acting toxins. Of these two theories, the latter appears to the authors to be more plausible and more in accordance with the results of experiments on the effect of toxins, more particularly pneumo-bacillin and diphtheria toxin, to which their investigations have been specially directed.

Behring[4] strongly opposes the view that the evil sequences which have been ascribed to anti-toxin are really due to the anti-toxin. He emphatically asserts that "diphtheria anti-toxin, the real and only substance of therapeutic significance in the anti-toxin serum, is absolutely harmless, and can never under any circumstances, either in man or beast, in the healthy or unhealthy organism, produce any toxic effect whatever." The evil sequences observed after anti-toxin treatment are entirely avoidable; they are due to albuminous bodies, salts, and accidental impurities, all of which have no therapeutic value. The living cells of the organism are found by experiment to be entirely uninfluenced by the anti-toxin; whatever changes occur in them are due to the serum, and are produced equally by serum taken from a healthy animal, a fact further proved by the identity of the changes, whatever the amount of anti-toxin in the serum. In order to eliminate partially or entirely the noxious substances mentioned, Behring has tried two methods of preparation of the anti-toxin. He first obtained serum containing very concentrated anti-toxin, and in this way hoped to minimise the dose of the serum required, and so to inject as little as possible of the noxious substances. A serious objection to this method was found to exist, for it was observed that these concentrated solutions quickly lose their strength by keeping. He has, therefore, devised a new method: a dried form of anti-toxin has been prepared, which is easily soluble in water. It is free from carbolic acid, contains no preservative, and is protected from micro-organisms by sealing in a closed vessel; one gramme of the dried preparation is equivalent to 5,000 normal units. In this way it is claimed that all evil sequences are avoided. Behring has further made some experiments on the absorption and excretion of anti-toxin. He finds that it does not form any chemical combination in the living organism, but circulates in the body fluids unaltered, and is gradually excreted in its unaltered condition. The rate of excretion, which was observed in the milk and urine of a goat, varies much, but it was found that an increased dose of anti-toxin caused disproportionate increase of excretion. Therefore, in attempting to prolong the period of immunity conferred by anti-toxin, it is better to repeat the dose than to increase the strength of the dose, for the repetition gives more certain prolongation of immunity.

We may allude here to Smirnow's[5] experiments on the production of artificial anti-toxin. He claims to have prepared an anti-toxin by electrolysis of a broth containing diphtheria toxin, and of such a strength that 0·5 c.cm. to c.o. c.cm. will cure guinea-pigs in 16 to 18 hours, after they have been infected with a virulent culture, killing control animals in 30 hours. He concludes that anti-toxin is oxidised toxin.

We will now refer to a series of articles recently collated and abstracted in the *Medical Chronicle*. First is that in which Spronck[6] discusses the vexed question of the identity of the Von Hofmann's pseudo-diphtheritic bacillus, which Loeffler and others maintain is distinct from the attenuated short diphtheria bacillus. Happily the doubtful cases where it can only be settled by inoculation experiments are not numerous. In 200 cases of angina and croup examined in Spronck's laboratory there were only four cases in which cultivation on serum yielded none but short bacilli. These short bacilli closely resembled one another in their mode of growth, but differed from the true bacillus in forming whiter, flatter, and more moist colonies when grown on Loeffler's serum, growing more abundantly on gelatine, never rendering bouillon markedly acid, and in other smaller particulars—in fine, they exhibited all the characteristics of the pseudo-diphtheritic bacillus of German observers, with the exception that two out of the four specimens proved pathogenic to guinea-pigs, the others, when injected in doses of 1—3 c.c., producing neither local nor general symptoms. In doses of 2 to 3 c.c.m. local œdema was marked, and there supervened loss of appetite, roughness of coat, &c., from which recovery was complete in two months. Spronck found that these latter results were not modified, whether the animals were treated before or after inoculation, by strong doses of anti-diphtheritic serum; he therefore concluded that these short bacilli, which absolutely correspond to the short variety of diphtheritic bacilli in Martin's classification, and, leaving aside their virulence, possess all the characters ascribed to the pseudo-diphtheritic bacillus of Hofmann, are not in reality true diphtheria bacilli. When cultivated for eight months, these bacilli lose their virulence, and are identical with Hofmann's bacillus. But Roux and Yersin have shown that, starting from a virulent diphtheritic bacillus, it is possible to also obtain a bacillus indistinguishable from that of Hofmann, hence Spronck considers that the pseudo-bacillus of Hofmann is sometimes true diphtheria bacillus, and at other times is not. It is worthy of note that the doubtful cases are not numerous, for in Spronck's laboratory in the 200 cases there were only these four in which serum cultures yielded none but short bacilli. E. A. Peters[7] bases a paper on the examination of 137 cases, and endeavours to show that two varieties of the diphtheria bacillus exist, and that these two forms never pass into each other, even when cultivated two years, the long form, and the short form. The short form on cultivation readily lost its virulence and became smaller, and then somewhat resembled Hofmann's bacillus. This latter organism was never met with in normal throats, but occurred in various catarrhal conditions, and frequently in cases of diphtheria associated with typical diphtheria bacilli. Its growth in gelatine never showed the clear acidity characteristic of the diphtheria bacillus. A long non-pathogenic bacillus, closely resembling the long Klebs-Loeffler bacillus, was obtained from a case of chronic faucial catarrh.

Cobbett and Phillips[8] divide the bacilli they obtained in 18 cases into virulent and non-virulent forms, the latter being again divided into those that resemble the virulent form in producing acidity when grown in glucose broth, and those that do not. When stained with Loeffler's methyl blue the acid-forming non-virulent forms showed great similarity to typical Klebs-Loeffler bacilli, *i.e.*, with a division into several deeply-stained segments, separated by narrow unstained or faintly stained intervals.

False membranes were produced by Roger and Bayeux[9] in rabbits by the intra-tracheal injection of a solution of pure toxin. As those in which false membranes formed showed no signs of general toxæmia, while those in which no membrane formed died, they conclude that the formation of false membrane showed a certain power of resistance to the poison which spends itself in causing local disturbance.

W. H. Park,[10] speaking in regard to the question of mixed infection in diphtheria, has stated that cultures from the blood of those dying early in diphtheria are usually sterile; when there was a high temperature septicæmia is generally formed. When the lungs showed lesions, diphtheria bacilli are always present in the blood, and streptococci are also found.

[1] Dublin Journal, Jan. 1, 1897. [2] Lancet, May 15, 1897. [3] Med. Week, Mar. 15, 1897. [4] Fortsch. der Med., Jan. 1897; epit. B.M.J., Feb. 6, 1897. [5] Arch. des Sciences Biolog. de l'Instit. Imp. de Méd. expér. à St. Petersbourg, Tome IV., No. 5; Epit. B.M.J., Feb. 27. [6] Le Sem. Med., Aug. 12, 1896. Med. Chron., Jan., 1897. [7] Journ. of Path. and Bact., Dec., 1896 (from the bacteriological laboratory of Guy's Hospital); Med. Chron., loc. cit. [8] Journ. of Path. and Bact., Dec., 1896 (from the Cambridge Path. Lab.); Med. Chron., loc. cit. [9] "Experim. Diph.," Soc. de Biol.; Presse Méd., Mar. 17, 1897; Journ. of Larynx, May, 1897. [10] New York Med. Journ., Oct 31, 1896.

### DISEASES OF CHILDREN.

(*Continued from page* 286.)

**The Specific Fevers and their Complications.**—Dr. Edward Mackey[1] has recorded two cases of measles with fatal complications of a somewhat unusual type. The first patient, a boy of ten years, with a fully-developed measles rash, suddenly became unconscious, and a few hours later he was found to be comatose, with stertorous breathing, insensible corneæ, dilated pupils, retracted head, spastic rigidity of limbs, and contracture of the fingers and toes. He died the same day, the temperature rising to 105 deg. Fahr. The necropsy revealed signs of intense cerebral hyperæmia without any definite cause. The writer suggests that toxins might have been circulating in the blood and overwhelmed the nerve centres. His second case was an infant of fifteen months, in whom the onset of measles was marked by a slight general convulsion. Ten days later œdema appeared in the right foot and left hand, which increased rapidly in the latter, with ecchymoses about the elbow, and vesicles on the wrist. The diagnosis was septic phlebitis with thrombosis, and the case terminated fatally a few days later. Dr. Lucas Benham[2] has recorded a case of mumps with cerebral symptoms and high temperature. In reviewing the subject he finds that the nervous phenomena associated with mumps fall into three classes, namely, (*a*) high fever and delirium, (*b*) insanity, and (*c*) paralysis. Such symptoms are usually induced by exposure to cold during convalescence from an ordinary attack, and in the majority of cases they subside under simple treatment.

Revilliod[3] reports a case of paralysis following mumps in a boy of seven years. Weakness of the legs was followed by dysphagia and rapid emaciation. During the illness all the limbs became affected, and, in addition, the following cranial nerves were involved, namely, the sixth on both sides, the left facial, the right hypoglossal, and part of the spinal accessory. The sphincters, the special senses, general sensation, and the vaso-motor system were intact. The symptoms were in many respects very similar to those of diphtheritic paralysis, but this could be excluded. Under the use of strychnine, massage, &c., the boy rapidly recovered, and was quite well in six weeks.

Brasch[4] has recorded a case of atypical scarlatina, with aphasia of toxic origin. The patient was a girl of four years, who, towards the end of the first week of an attack of scarlatina lost the power of speech. Other nervous symptoms were present, but these gradually subsided, and the power of speech was restored at the end of six weeks. A point in the early diagnosis of measles is referred to by Koplik,[5] who finds that before the rash appears the buccal mucous membrane and the inside of the lips present a distinct eruption of small irregular spots of a bright red colour. In the centre of each spot there is a minute bluish-white speck, which is pathognomonic of commencing measles.

**Cerebral Lesions and Intestinal Disorders.**—In a clinical lecture Dr. Marfan[6] points out that certain meningeal and cerebral lesions may be the result of toxi-infection of gastro-intestinal origin. He has met with several cases of acute gastro-enteritis, associated with convulsions, and terminating in the development of a syndrome pointing to a chronic intra-cranial lesion, such as hydrocephalus, idiocy, hemiplegia, epilepsy, &c. He points out that such a view of the toxic action of the intestinal contents is quite in accordance with the teachings of modern neuro-pathology.

**Tetany.**—In the ordinary form of tetany, Hauser[7] finds that rickets is an important predisposing and gastro-intestinal disturbance an exciting cause. Treatment directed to the latter will often rapidly cure an attack. There is also a form of latent tetany of which severe laryngeal spasm is the most prominent symptom. This subject has also been exhaustively discussed by Oddo,[8] who accepts the view that the essential cause is a form of toxæmia due to the absorption of poisonous bodies produced in the intestinal canal. He assumes the existence of two conditions—(1) a special form of perverted digestion, associated usually with dilatation of the stomach, and (2) a special vulnerability of the nervous system. The commonest symptoms are spasm of the extremities and of the larynx, while less frequently retraction of the head and squinting are present. He recommends the employment of calomel in doses of three-quarters of a grain or more, according to the age of the child, with which may be combined benzo-naphthol or bismuth if the motions are offensive or fermenting. Lavage of the stomach is useful, and it is important to ascertain and correct any defect or excess of the acids in the gastric contents. Hot baths, warmth, and avoidance of all excitement are important measures in quieting the nervous system, with which may be combined chloral administered per rectum in four-grain doses four times a day for an infant. If laryngeal spasm becomes alarming, he advises the application of a hot sponge to the front of the neck, followed by the inhalation of chloroform, administered very gradually, as it may have a tendency at first to increase the spasm.

[1] Brit. Med. Jour., Dec. 19, 1896. [2] The Lancet, Jan. 30, 1897. [3] Rev. Med. de la Suisse Romande, Dec. 20, 1896, and Ep. Brit. Med. Jour, Feb. 27, 1897. [4] Berl. Klin. Wochensch, Jan. 11, 1897, and the Med. Rec., Feb. 13, 1897. [5] Arch. of Ped., Dec. 1896, and Ep. B.M.J., March 13, 1897. [6] The Med. Week, Aug. 21, 1896. [7] Berl. Klin. Wochens, 1896, No. 35, and Am. Jour. Med. Sci., Nov., 1896. [8] Rev. de Med., June—Sept., 1896, and Ep. B.M.J., Nov. 21, 1896.

